# Exploring the phytochemicals, antioxidant properties, and hepatoprotective potential of *Moricandia sinaica* leaves against paracetamol-induced toxicity: Biological evaluations and in Silico insights

**DOI:** 10.1371/journal.pone.0307901

**Published:** 2024-10-09

**Authors:** Shaza H. Aly, Abdulla M. A. Mahmoud, Sherif S. Abdel Mageed, Eman F. Khaleel, Rehab Mustafa Badi, Eslam B. Elkaeed, Rabab Ahmed Rasheed, Mahmoud A. El Hassab, Wagdy M. Eldehna

**Affiliations:** 1 Department of Pharmacognosy, Faculty of Pharmacy, Badr University in Cairo (BUC), Badr City, Egypt; 2 Pharmacology and Toxicology Department, Faculty of Pharmacy, Badr University in Cairo (BUC), Badr City, Cairo, Egypt; 3 Department of Medical Physiology, College of Medicine, King Khalid University, King Khalid University, Asir, Saudi Arabia; 4 Department of Pharmaceutical Sciences, College of Pharmacy, AlMaarefa University, Riyadh, Saudi Arabia; 5 Department of Histology & Cell Biology, Faculty of Medicine, King Salman International University, South Sinai, Egypt; 6 Department of Medicinal Chemistry, Faculty of Pharmacy, King Salman International University (KSIU), South Sinai, Egypt; 7 Department of Pharmaceutical Chemistry, Faculty of Pharmacy, Kafrelsheikh University, Kafrelsheikh, Egypt; 8 Department of Pharmaceutical Chemistry, Faculty of Pharmacy, Pharos University in Alexandria, Alexandria, Egypt; G.B. Pant National Institute of Himalayan Environment (GBP-NIHE), INDIA

## Abstract

Thirteen components were identified in the methanol extract of *Moricandia sinaica* leaves (MSLE) through analysis utilizing HPLC-ESI-MS/MS., including flavonoids, anthocyanins, phenolic acids, and fatty acids. The methanol extract of *M*. *sinaica* leaves contained total phenolics and flavonoids (59.37 ± 2.19 mg GAE/g and 38.94 ± 2.72 mg QE/g), respectively. Furthermore, it revealed *in vitro* antioxidant properties as determined by the DPPH and FRAP assays, with respective IC_50_ values of 10.22 ± 0.64 and 20.89 ± 1.25 *μ*g/mL. The extract exhibited a notable hepatoprotective effect in rats who experienced paracetamol-induced hepatotoxicity. When a dose of 250 mg/kg was given, there was a 52% reduction in alanine transaminase and a 30% reduction in aspartate transaminase compared to the group with the disease. Furthermore, it demonstrated a 3.4-fold, 2.2-fold, and 2.6-fold increase in superoxide dismutase, non-protein sulfhydryl, and glutathione peroxidase, respectively. In addition, it demonstrated a 68% decrease in lipid peroxide levels compared to the group with paracetamol-induced condition. The verification was conducted using a histological study, which identified improved liver histology with a small number of distended hepatocytes. Moreover, *in silico* studies focused on the enzymes NADPH oxidase, butyrylcholinesterase, and tyrosinase as the targets for the major compounds. In conclusion, MSLE showed promising hepatoprotective and antioxidant activities due to its richness in antioxidant metabolites.

## 1. Introduction

The liver is a vital organ that performs several functions, including the storage of vitamins, iron, and glycogen, as well as the metabolism of carbohydrates, lipids, and proteins. Moreover, the liver has a vital function in eliminating many external compounds from the body by facilitating their removal through processes like reduction, hydrolysis, oxidation, and conjugation. Moreover, it is accountable for the synthesis of 90% of the plasma proteins and the production of bile [1–3].

Chronic liver illnesses remain a substantial worldwide health issue, impacting more than 10% of the global population [4]. Up to 40% of people with cirrhosis may not show any symptoms and can remain without symptoms for long periods of time. However, when complications like variceal hemorrhage, ascites, or encephalopathy occur, there is often a progressive decline that can result in mortality or the need for a liver transplant [5].

The liver’s hepatocytes employ various hepatic defense mechanisms to protect against oxidative damage. These mechanisms include the synthesis of reduced glutathione (GSH), a natural antioxidant, and superoxide dismutase (SOD), an enzyme that converts the superoxide radical into molecular oxygen and hydrogen peroxide, thereby facilitating detoxification [6–10].

The antioxidant, antibacterial, anti-inflammatory, and cytotoxic capabilities of natural sources are significant [11–13]. The existence of secondary metabolites in their composition makes their ability to contribute to antioxidant defense and redox signaling possible. These secondary metabolites include flavonoids, phenolic acids, terpenoids, and volatile oils. These compounds act as scavengers of free radicals [14–18]. Various hepatoprotective compounds derived from plants function as effective pharmaceuticals and dietary supplements. Hepatoprotective drugs produced from natural sources are of great significance for such agents as glycyrrhizin, ellagic acid, and silymarin [19, 20].

*Moricandia* is a genus belonging to the family Brassicaceae and comprises approximately eight species that exhibit various biological activities such as cytotoxicity, antioxidant properties, and anti-inflammatory effects [21–23]. Regarding the health benefits, leaves of *M*. *arvensis* are frequently incorporated into traditional Tunisian cuisine, and *M*. *sinaica* has been utilized traditionally as a treatment for both pain and syphilis [24].

*Moricandia sinaica* (Boiss.) is a herbaceous plant that grows for one year and is endemic to the Mediterranean region of Europe and America. The species is predominantly cultivated globally as decorative plants, exhibiting flowers in a variety of shades, including violet, purple, and white [25]. Currently, there are limited studies on the biological activities or the chemical composition of *M*. *sinaica*. A previous study on the leaves of *M*. *sinaica* by Aly et al. (2023) reported the chemical composition of the volatile oil and *n*-hexane extract of the leaves; the results revealed their richness with aliphatic hydrocarbons. Additionally, the primary constituents of the hexane extract include *α*-amyrin, *γ*-sitosterol, *β*-amyrin acetate, and *α*-tocopherol. Conversely, the primary constituents of the essential oil were predominantly monoterpenes and sesquiterpenes. Besides, the oil showed promising antioxidant and cytotoxicity *in vitro* [21]. Another study reported the anti-inflammatory, analgesic, and antipyretic activities *in vivo* of the water extract of *M*. *sinaica* along with the characterization of its polyphenols where the extract was predominantly composed of flavonoid, namely isorhamnetin, quercetin, and kaempferol [24].

We conducted a study to investigate the potential antioxidant and hepatoprotective properties of *M*. *sinaica* leaves extract (MSLE) when exposed to a paracetamol-induced toxicity model. This research was prompted by the increased demand for new hepatoprotective medicines obtained from natural sources. In addition, we performed a phytochemical analysis of the extract components using HPLC/ESI/MS/MS in combination with molecular docking research.

## 2. Materials and methods

### 2.1. Plant material

*Moricandia sinaica* Boiss. (Brassicaceae) leaves were collected in February 2022 from South Sinai, Egypt, 27°57’43.2"N and 34°16’16.7"E. Dr. Mohammed El-Gebaly, a professor in botany department at the National Research Centre in Giza, Egypt, identified and verified the plant with gratitude. The voucher specimens, labelled BUC-PHG-MS-10, were submitted to the Pharmacognosy Department of the Faculty of Pharmacy, Badr University in Cairo.

### 2.2. Preparation of the plant extract

The air-dried leaves of *Moricandia sinaica* (100 g) were initially extracted with *n*-hexane. The leaves were soaked in n-hexane (3 x 1L), then the liquid was separated by filtration and evaporated under vacuum at 40°C until all the solvent evaporated, resulting in the production of 2.41 g of the *n*-hexane extract. The defatted leaves were macerated in 80% methanol (3 x 1L) and then filtered. The filtrate was subjected to drying using a Rotary evaporator (Hei-VAP Value, Heidolph) at a temperature of 45°C. The collected dried residue (MSL) (17.63 g) was kept in the refrigerator till used in the study [26].

### 2.3. HPLC-ESI-MS/MS analysis

The phytochemical characterization of the methanol extract of *M*. *sinaica* leaves was performed by employing HPLC coupled with ESI-MS following a recently published method [27]. Briefly, the extract was diluted in HPLC-grade methanol (100 μg/mL), filtered with a 0.2 μm membrane disc filter, and injected in 10 μL. The Acquity HPLC apparatus (Waters^®^, Milford, MA USA) utilized a reversed-phase Acquity UPLC-BEH C_18_ column (1.7 μm particle size, 2.1 x 50 mm). Over a 35-minute run, with a gradient of acidified water and methanol with 0.1% formic acid, mobile phase elution flow was increased to 0.2 mL/min. An XEVO TQD triple quadruple instrument was used to conduct ESI-MS in both positive and negative ion acquisition modes. The triple quadrupole mass spectrometer XEVO TQD (Waters Corporation, Milford, MA, USA) was utilized for mass spectrometric analysis under these conditions: Edwards^®^ vacuum pump (Chandler, AZ, USA) delivers 30 eV cone voltage and 3 kV capillary voltage at 150° source and 440° desolvation temperatures. Maslynx 4.1 observed mass spectra in the ESI region between 100–1000 m/z, and the fragmentation pattern and mass spectra were used to tentatively identify the peaks with reported data.

### 2.4. Total phenolic and flavonoid content

The Folin-Ciocalteu and AlCl_3_ spectrophotometric techniques were used to measure the extracts’ total phenolic and flavonoid content. Our previous published work provides the experimental methods [27]. The total phenolic concentration was quantified in milligrams of gallic acid equivalents (GAE) per gram of dry extract [28]. The total flavonoid concentration was evaluated as mg quercetin equivalents (QE) per gram of dry extract [29].

### 2.5. *In vitro* antioxidant assays

#### 2.5.1. DPPH and FRAP assays

The DPPH radical scavenging activity and FRAP ferric-reducing antioxidant power assays were performed based on previously published methods [30–32]. All measurements were done three times and averaged S1 and S2 Figs.

### 2.6. *In vivo* hepatoprotective activity

#### 2.6.1. Animals

The National Research Centre in Cairo, Egypt, provided 30 mature male Wistar albino rats weighing 200–220 g and ten weeks old for the study. In regulated laboratory circumstances, rats were housed at 25 ± 2°C, 40–60% humidity, and a 12-hour light-dark cycle. Rats were fed a regular diet and given water freely. The rats were lab-acclimated for a week before the experiment began. Each animal received a temporary random identifier within its respective weight range category. Based on their placement on the rack, cages were assigned numerical labels. For each group, a cage was randomly chosen from the entire pool of cages. animals were then selected from each weight range category and assigned their permanent numerical identifiers within the cages. Subsequently, the cages were randomized within the designated exposure group. The Research Ethics Committee of Badr University’s in Cairo, Faculty of Pharmacy authorized the study protocols (PG-117-A) in accordance with the guidelines of the National Institutes of Health for the proper care and use of laboratory animals (NIH Publication No. 85–23, revised 2011).

In our study, we have prioritized the well-being of animals involved in scientific research to uphold the credibility and dependability of our findings. Through diligent monitoring and implementing humane practices, we have consistently evaluated the health conditions of rats. Our assessments, recorded in a comprehensive scoresheet, encompassed various factors such as physical appearance, weight changes, behavior, and locomotion. The majority of rats demonstrated normal scores, indicating overall good health [33].

#### 2.6.2. Experimental design

In this study, a total of 30 rats were randomly allocated into five different weight groups according to a computer-generated randomization table (www.randomization.com) (n = 6 per group) as follows: Group I: served as a normal control and received 10% Tween 80 (10 mL/kg, po) for 14 days. Group II: received MSLE (250 mg/kg, p.o.) as a drug alone group [34]. Group III: received 10% Tween 80 *via* oral route for 14 days and then received PAR (2 g/kg, p.o.) [35, 36] suspended in 10% Tween 80 1 hour after the last dose, serving as the disease group. Group IV: received MSLE (250 mg/kg, p.o.) for 14 days and then received PAR (2 g/kg, p.o.) suspended in 10% Tween 80 1 hour after the last dose, serving as the treatment group. Group V: received (N-Acetylcysteine (NAC) at a dose of (100 mg/kg, po) dissolved in 10% Tween 80 and followed by PAR (2 g/kg, p.o.) suspended in 10% Tween 80 one hour after the last dose, serving as the standard group [36]. Power analysis method (power = 0.8, α = 0.05) was conducted to estimate group size.

#### 2.6.3. Blood sampling and tissue preparation

After PAR intoxication, blood samples were obtained 4 hours later and then subjected to centrifugation at a speed of 3000 rpm for 5 minutes. The specimens were preserved at a temperature of -20ºC for subsequent examination. The animals were administered thiopental (50 mg/kg) for anesthesia after a period of 24 hours and then sacrificed by decapitation by well-trained personnel according to AVMA Guidelines for the Euthanasia of Animals: 2020 Edition [37]. The liver was expeditiously excised, quantified, rinsed with frigid saline solution, and partitioned into two segments. The initial portion was combined with phosphate buffer saline (0.1 M PBS, pH 7.4) and subjected to centrifugation at a rate of 10,000 revolutions per minute for a duration of 30 minutes at a temperature of 4ºC. The liquid portion of the mixture was subsequently preserved at a temperature of -80ºC for future biological analyses. The second portion of the liver was stored in a solution of 10% neutral buffered formalin for the purpose of conducting histological analysis. During the analysis of these measurements, the investigators were blinded to sample identity and an independent experimenter performed sample coding and decoding.

#### 2.6.4. Biochemical analyses

*2*.*6*.*4*.*1*. *Plasma biochemical parameters*. The concentrations of AST and ALT in the blood samples were determined using commercially available kits from Bio-Diagnostic (Giza, Egypt) and a UV-visible spectrophotometer (V630; JASCO, Tokyo, Japan) at a wavelength of 505 nm. The data were evaluated using standard methods [38].

*2*.*6*.*4*.*2*. *Tissue biochemical parameters*. *2*.*6*.*4*.*2*.*1*. *Estimation of lipid peroxidation levels*: Thiobarbituric acid (TBA) was used to test lipid peroxidation in homogenized liver tissue supernatant. The malondialdehyde (MDA) production can be measured based on this method. The TBA-MDA complex absorbance was measured at 530 nm, and liver MDA concentration was reported in nmol/mg protein [39].

*2*.*6*.*4*.*2*.*2*. *Estimation of superoxide dismutase activity*: The activity of superoxide dismutase (SOD) was determined using spectrophotometric measurement, as described by Robak et al. [40]. This method depended on the generation of superoxide ions by the oxidation of xanthine and oxygen by xanthine oxidase, resulting in the formation of uric acid and hydrogen peroxide. Superoxide anions convert nitroblue tetrazolium (NBT) into NBT-diformazan, which absorbs at a wavelength of 550 nm. Superoxide dismutase (SOD) was tested for its ability to reduce superoxide ions and prevent NBT-diformazan production. Reduced NBT-diformazan production measures Superoxide Dismutase (SOD) activity in the tested sample.

*2*.*6*.*4*.*2*.*3*. *Estimation of reduced glutathione levels*: The concentration of reduced glutathione (GSH) was determined based on [41, 42]. This approach relies on the interaction between DTNB [5,50-dithiobis-(2- nitrobenzoic acid)] and reduced glutathione (GSH), resulting in the creation of a yellow-colored molecule that exhibits its highest absorption at 412 nm. The GSH concentration was measured and expressed as nmol/mg protein.

*2*.*6*.*4*.*2*.*4*. *Estimation of antioxidant enzyme activities*: The Glutathione peroxidase (GPx) activity was evaluated using a colorimetric assay. Hydrogen peroxide is the substrate in this GSH-based test. The absorbance was measured at 420 nm, and GPx specific activity was reported as μmol GSH/mg protein [43].

#### 2.6.5. Histopathological examination

Liver tissue samples were dissected, then preserved in 10% neutral buffered formalin (NBF) and dried using increasing concentrations of alcohol. The liver samples were immersed in paraffin to create solid blocks, which were then cut into thin sections measuring 4–6 μm in thickness. To assess histology, these slices were stained with hematoxylin and eosin [44]. Ordinary light microscopy [Olympus, Japan] was used to capture images at X400 magnification.

### 2.7. Statistical analysis

The acquired data were displayed as the mean ± standard deviation (SD) using one-way analysis of variance (ANOVA) to examine the variations between the groups. Tukey’s t-test was then employed as a post hoc test. The results were regarded as statistically significant if P ≤ 0.05.

### 2.8. Molecular docking

The X-ray crystallographic structures of NADPH oxidase, butyrylcholinesterase, and tyrosinase were obtained from the Protein Data Bank (www.pdb.org) on 15 August 2023. The structures were downloaded using the corresponding IDs: 2CDU, 6ESJ, and 5M8Q [45–47]. All of the docking studies were carried out with the help of the Vina autodock applications [48, 49]. After ChemDraw sketched the ligands, they were converted to 3D structures by the Biovia Discovery Studio visualizer. The MGL tools were used to prepare both the ligands and receptors in the pdbqt format as a prerequisite for the Vina autodock. The seven identified major compounds were subjected to docking analysis in the active site of each target. The active site was defined beforehand based on the binding of the relevant co-crystalized ligand. Ultimately, the docking outcomes were observed and examined according to the docking scores.

## 3. Results and discussion

### 3.1. Chemical composition of MSLE

The *M*. *sinaica* leaves extract (MSLE) was subjected to analysis using HPLC-ESI-MS/MS to identify its phytoconstituents S3 Fig. The findings indicated the detection of thirteen phenolic components from various groups, including flavonoids, anthocyanins, and phenolic acids, in MSLE. Their MS properties and fragmentation pattern were compared to previous published data in order to make a tentative identification (Table 1). Among the identified secondary metabolites in MSLE, the flavonoid glycosides were found to be the most predominant class. The ion mass peaks at m/z 509.25, 577.35, 593.25, 535.25, 621.30 for the suggested molecular formulas C_23_H_24_O_13_, C_27_H_30_O_14_, C_28_H_32_O_14_, C_25_H_25_O_12_, C_32_H_27_O_13_, respectively, corresponding to five flavonoid glycosides; namely syringetin-3-O-glucoside, kaempferol-3,7-O-*bis*-α-L-rhamnoside, linarin, luteolin-O-diacetylhexoside, apigenin derivative (**6**, **7**, **9**, **11**, **13**). A previous study on the aqueous fraction of *M*. *sinaica* displayed a high percentage of polyphenols represented as flavonoid glycosides that are in correlation with our results, and the extract was reported for its anti-inflammatory activity [24]. Besides, Linarin, known as acacetin-7-O-*β*-D-rutinoside, was previously identified from *Isatis indigotica* (Brassicaceae) [50, 51].

**Table 1:** Characteristics of *M*. *sinaica* leaves extract (MSLE) phytoconstituents identified by HPLC-MS/MS in the positive and negative ionization mode.

No.	R_t_ (min)	Tentative identified compound	[M-H] ^¯^/[M+H] ^+^ (*m/z*)	MS^2^ fragments (*m/z*)	Compound Class	Molecular Formula	Ref.
1	0.69	Caffeic acid derivative	377.10	341	Phenolic acid	C_18_H_17_O_9_	[54]
2	3.45	Sinapic acid 3-O-glucoside	409.15 [M+Na] ^+^	-	Phenylpropanoid acid	C_17_H_22_O_10_	[55]
3	9.47	5,7-Dihydroxy-2’-methoxy-3’,4’-(methylenedioxy)isoflavone	327.30	179	Flavonoid	C_17_H_12_O_7_	[56]
4	10.89	Isorhamnetin	315.05	315, 300, 151	Flavonoid	C_16_H_12_O_7_	[57]
5	11.19	Trihydroxy Octadecenoic acid	329.10	211, 229, 171, 139, 99, 155	Fatty acid	C_18_H_34_O_5_	[54]
6	13.80	Syringetin-3-O-glucoside	-/509.25	-	Flavonoid glycoside	C_23_H_24_O_13_	[56]
7	13.83	Kaempferol-3,7-O-*bis*-alpha-L-rhamnoside	577.35	431, 285	Flavonoid glycoside	C_27_H_30_O_14_	[56]
8	15.63	3′,3′′′-Binaringen methyl ether	555.35	403, 237, 219, 151	Biflavonoid	C_31_H_24_O_10_	[58]
9	23.27	Linarin (Acacetin-7-O-β-D-rutinoside)	-/593.25	-	Flavonoid glycoside	C_28_H_32_O_14_	[59]
10	23.82	Cyanidin rutinoside	-/593.30	287, 434, 595	Anthocyanin	C_27_H_31_O_15_+	[60]
11	24.69	Luteolin-O-diacetylhexoside	-/535.25	490, 489, 285	Flavonoid glycoside	C_25_H_25_O_12_	[61]
12	26.98	Australisine A	-/607.25	286	Flavonoid	C_35_H_26_O_10_	[60]
13	28.12	Apigenin derivative	-/621.30	269	Flavonoid glycoside	C_32_H_27_O_13_	[62]

**Fig 1:**
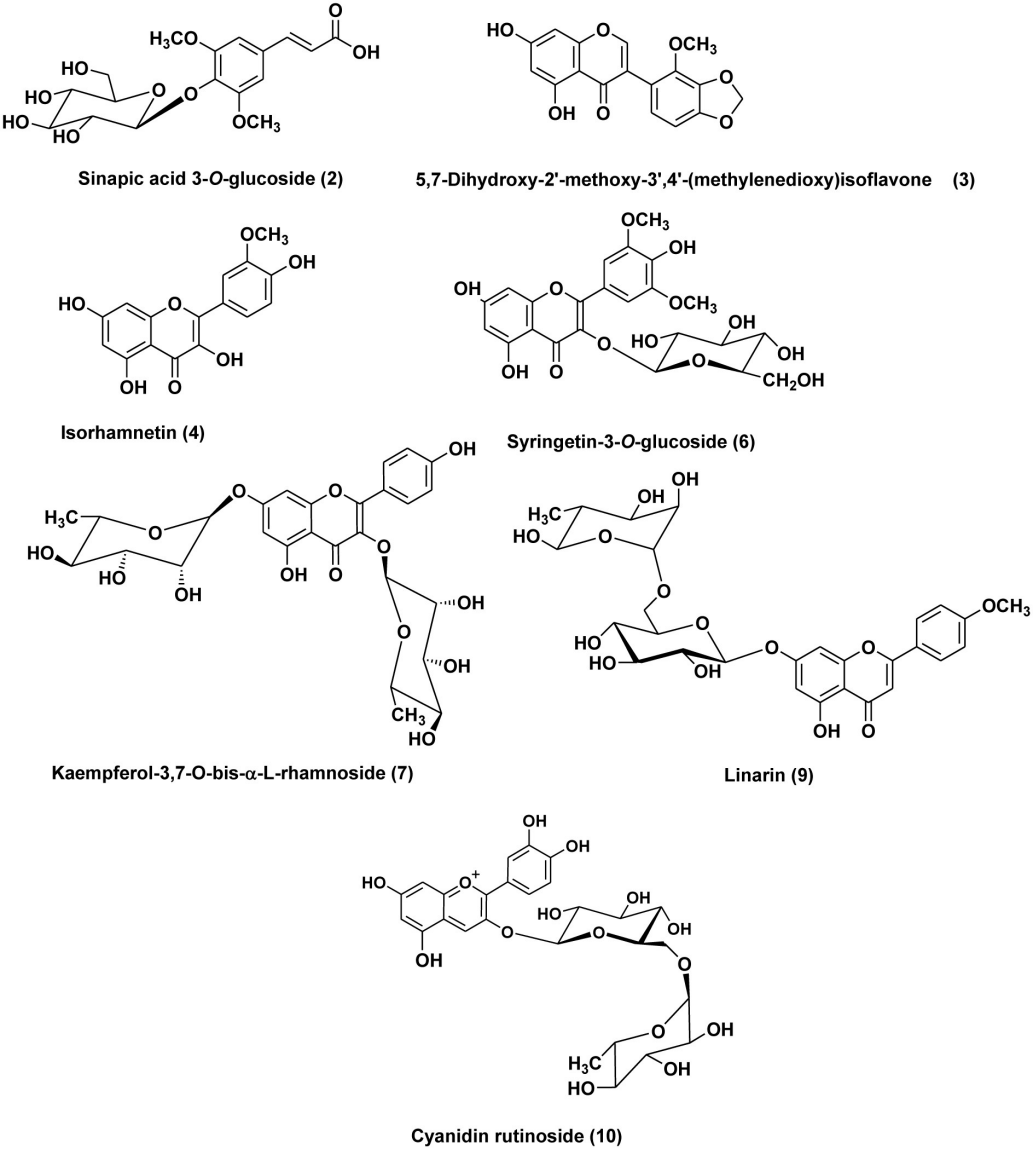
The chemical structures of major compounds of the *M*. *sinaica* leaves extract (MSLE) identified by HPLC-MS/MS.

Besides, three ion peaks values at *m/z*327.30, 315.05, and 607.25 with molecular formulas C_17_H_12_O_7_, C_16_H_12_O_7_, and C_35_H_26_O_10_, respectively were tentatively identified as flavonoid aglycones **(3, 4, 12)** 5,7-dihydroxy-2’-methoxy-3’,4’-(methylenedioxy) isoflavone, isorhamnetin, and australisine A. Biflavonoid was tentatively characterized as 3’,3‴-binaringen methyl ether (**8**), The compound displayed a deprotonated ion [M-H]^-^ at *m/z* 555.35, and its MS2 pattern matched the previously documented data for biflavonoid 3’,3‴-binaringen methyl ether [52]. Moreover, anthocyanin is characterized as cyanidin rutinoside with a protonated ion peak value at *m/z* 593.30 in (**10**) that may improve the hepatoprotective potential of the extract through its antioxidant effect [53]. Phenolic acid and phenylpropanoic acid in MSLE are represented as caffeic acid derivative **(1)** and sinapic acid 3-*O*-glucoside **(2).** The chemical structures of the major components in MSLE are depicted in (Fig 1). Based on our results, we were encouraged to conduct an *in vitro* antioxidant and i*n vivo* hepatoprotective study due to the identification of bioactive secondary metabolites in the methanol extract of *M*. *sinaica* leaves.

### 3.2. Total phenolics (TP) and total flavonoids (TF)

The Folin-Ciocalteu and AlCl_3_ methods, which are spectrophotometric techniques, were employed to quantify the total phenolics and total flavonoids in the MSLE. Polyphenolics, a type of secondary metabolites, possess numerous biological activities such as antioxidant, cytotoxic, antidiabetic, and anti-inflammatory effects [63–65]. The methanol extract of M. sinaica leaves exhibited total phenolics and total flavonoid contents corresponding to 59.37 ± 2.19 mgGAE/g and 38.94 ± 2.72 mgQE/g, respectively. This suggests it is a suitable candidate for antioxidant and hepatoprotective applications.

### 3.3. *In vitro* antioxidant potential of MSLE

Regarding the antioxidant potential of MSLE, it was estimated using two techniques DPPH and FRAP assays (Table 2). The results revealed its potent activity with an IC_50_ value of 5.78 ± 0.67 *μ*g/mL and 17.35± 1.28 *μ*g/mL in comparison to ascorbic acid standard with an IC_50_ value of 10.22 ± 0.64 and 20.89 ± 1.25 *μ*g/mL in DPPH and FRAP assays, respectively.

**Table 2:** Antioxidant potential, TPC and TPC of *M*. *sinaica* leaves extract (MSLE).

Extract	DPPH assay IC_50_ (*μ*g/mL)	FRAP assay IC_50_ (*μ*g/mL)	Total phenolics (mg/g gallic acid equivalents) mgGAE/g	Total flavonoids (mg/g quercetin equivalents) mgQE/g
MSLE	5.78 ± 0.67	17.35 ± 1.28	59.37 ± 2.19	38.94 ± 2.72
Ascorbic acid	10.22 ± 0.64	20.89 ± 1.25	-	-

Values are mean ± SEM, n = 3, IC_50_: Inhibitory concentration 50%.

Earlier studies have examined the antioxidant properties of plants from the genus *Moricandia*. The ethyl extract of *M*. *arvensis* shown significant ability to remove DPPH radicals, with an IC_50_ value of 171.9 ± 1.0 *μ*g/mL. Additionally, it effectively protected linoleic acid from peroxidation in *β*-carotene bleaching tests, with an IC_50_ value of 35.69 ± 2.30 *μ*g/mL [66–68].

### 3.4. *In vivo* hepatoprotective activity of MSLE

#### 3.4.1. Effects on liver stress markers

**Fig 2:**
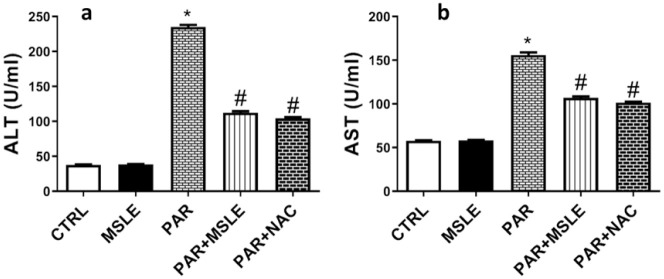
The effect of *M*. *sinaica* leaves extract (MSLE) identified on serum liver function markers in PAR-induced liver toxicity in rats. (a) Alanine aminotransferase, (ALT) (b) Aspartate aminotransferase (AST). Data are presented as mean ± SD (n = 6); * p < 0.05 vs. control rats; # p < 0.05 vs. PAR-treated rats using Tukey’s post hoc test.

The administrated dose of PAR resulted in a substantial elevation of liver enzymes ALT and AST by 6-fold (*P* < 0.0001) and 2.7-fold (*P* < 0.0001), respectively, in contrast to the control group. Preliminary treatment with MSLE significantly mitigated liver injury caused by PAR, as shown in (Fig 2) by decreasing ALT and AST levels by (*P* < 0.0001; 52%) and (*P* < 0.0001; 30%), respectively, in comparison to PAR group. Notably, MSLE showed a similar effect as the standard drug NAC (N-Acetylcysteine), there was no notable difference observed between the two groups.

#### 3.4.2. Effects on oxidative stress markers

**Fig 3:**
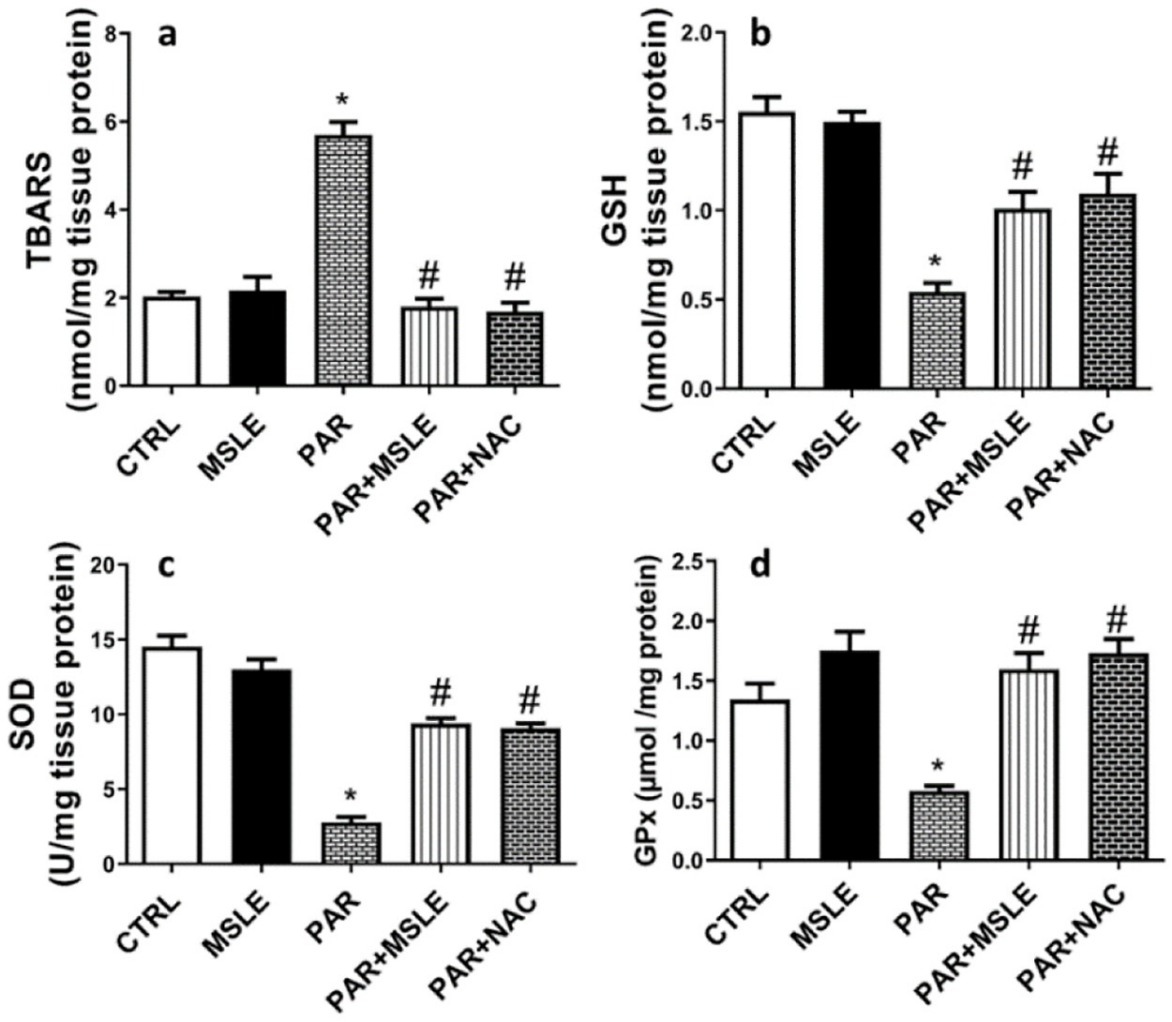
The effect of *M*. *sinaica* leaves extract (MSLE) identified on oxidative stress markers in PAR-induced liver toxicity in rats. (a) TBARs, (b) GSH, (c) SOD, and (d) GPx. Data are presented as mean ± SD (n = 6); * p < 0.05 vs. control rats; # p < 0.05 vs. PAR-treated rats using Tukey’s post hoc test.

The administration of paracetamol resulted in a significant rise in the formation of reactive oxygen species (ROS), which subsequently led to a substantial elevation in the level of thiobarbituric acid reactive substances (TBARS) (*P* < 0.0001; 2.9-fold) relative to the control group. This was confirmed by a substantial reduction in the activity of SOD (*P*<0.0001; 5.4-fold), GSH (*P* <0.0001; 3-fold), and GPx (Glutathione peroxidase) enzyme activity (*P* < 0.0001; 57%) (Fig 3). Rats treated with MSLE demonstrated notable antioxidant activity and effectively restored the redox imbalance induced by PAR, as compared to the PAR group that demonstrated by lower levels of lipid peroxide (*P* <0.0001; 68%) and elevated levels of SOD (*P*< 0.0001; 3.4-fold), GSH (P = 0.0007; 2.2-fold), and GPx activity (*P* <0.0001; 2.6-fold). *M*. *sinaica* leaves extract had similar effects as NAC, with no statistically significant distinctions observed between the two groups.

The excessive consumption of Paracetamol (PAR) can be harmful to the liver when used within the recommended therapeutic dose for alleviating mild pain and headaches, it undergoes biotransformation and is eliminated from the body as harmless conjugates of sulphate and glucuronic acid [69]. The overdose of PAR can cause hepatic necrosis, liver lesions, and kidney injury, and may even result in fatalities in both humans and experimental animals [70]. Hepatic enzymes (ALT and AST) will be released from the injured liver into the bloodstream, increasing their levels in blood tests [71]. Additionally, the oxidative stress and ATP depletion caused by mitochondrial permeability transition may be the cause of the hepatic damage brought on by PAR [72]. In our study, the MSLE (250 mg/kg) was used to explore the extract’s potential for protection against PAR-induced toxicity in rats. Compared to the group that received only PAR treatment, the group that received MSL after PAR therapy had significantly lower levels of AST and ALT. The results align with the prior study conducted by Laraba et al. (2022), who demonstrated the hepatoprotective effects of *n*-butanol extract from *M*. *arvensis* in cases of doxorubicin-induced toxicity. The extract was administered at doses of 50 mg/kg and 100 mg/kg. Compared to the DOX-treated rats, it significantly reduced the amount of TBARS, elevated GSH, and improved GPx activity. Evidence of the liver’s hepatoprotective efficiency was demonstrated by the preservation of its architecture and the reduction of structural and functional changes [73]. Another study showed the protective effects of *M*. *sinaica* methanol and *n*-butanol extracts (100 mg/kg and 200 mg/kg) regarding the oxidative stress on the kidney and heart through carbon tetra chloride (CCl_4_)-induced nephron and cardiotoxicity. Also, it demonstrated a protective effect for MDA and NPSH (non-protein sulfhydryl) levels [74].

The antioxidant and hepatoprotective potency of MSLE would be correlated with the presence of flavonoids as the major identified components (Table 1) that were reported for their hepatoprotective and antioxidant effects [75, 76], besides the high TPC and TFC (Table 2). Syringetin-3-O-glucoside was reported for its antioxidant properties [77, 78]. Another flavonoid, kaempferol, showed a significant decrease in oxidative stress and lipid peroxidation as well as an increase in antioxidative defense activity [79]. Another investigation on kaempferol glycosides namely, kaempferol 3-O-rutinoside and kaempferol 3-O-glucoside, showed that they increased total protein levels and prevented the CCl_4_-induced elevations in AST, ALP, and MDA levels in the serum, and revealed normal catalase (CAT) and SOD activity and had greatly recovered GSH levels [80]. Furthermore, apigenin, a flavonoid found in MSLE, has been shown to possess hepatoprotective properties by mitigating liver damage through the reduction of oxidative stress and inflammation [81]. Eventually, the presence of bioactive secondary metabolites in the methanol extract of *M*. *sinaica* leaves clarified that providing MSLE to rats exposed to PAR-induced toxicity considerably reduced liver damage and eased fibrosis and was thus thought to be a possible herbal remedy for treating liver injury.

### 3.5. Histopathological investigation of the liver

**Fig 4:**
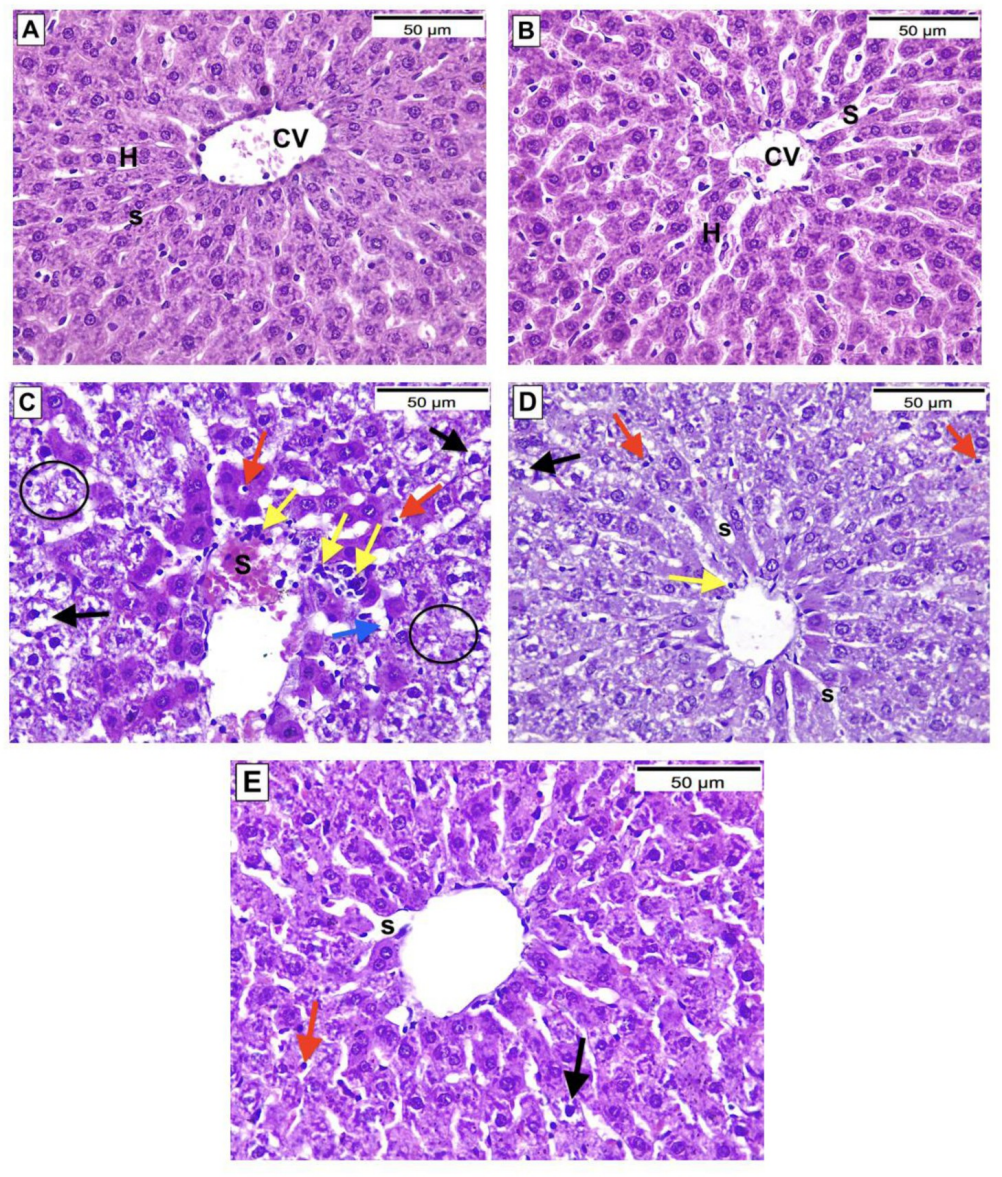
Hematoxylin & eosin-stained sections from livers of all study groups, magnification X400. (A & B) Group I (control) & II (MSLE) respectively. (C) Group III (PAR). (D) Group IV (Treatment with MSLE). (E) Group V (Treatment with NAC). C.V: Central vein, H: Hepatocytes, S: Blood Sinusoids, Black arrows: Ballooning of hepatocytes, Red arrows: Apoptotic hepatocytes, Blue arrows: Lipid droplets, Yellow arrows: Inflammatory cells. Circles: Lobular necrosis.

Histopathological assessment and grading of liver tissue injury were based upon previous study [82] as shown in (Table 3). Group I (control) & II (MSLE) showed normal liver histology with acidophilic hepatocytes arranged in regular cords radiating from the central vein [Grade 0]. Group III (PAR) showed disorganized liver architecture, severely degenerated hepatocytes with ballooning [Garde II], apoptotic cells, cytoplasmic lipid droplets [Grade III], focal lobular necrosis [Grade IV], markedly congested sinusoids, and heavy inflammatory cellular infiltrates. Group IV, which was treated with MSLE, showed improved liver histology with few ballooned hepatocytes [Grade II], fewer apoptotic cells, and mildly congested sinusoids. Group V treated with NAC showed improved liver histology with scarce ballooned hepatocytes [Grade II], rare apoptotic cells, and mild sinusoidal congestion (Fig 4).

**Table 3:** Histological grading of liver injury.

Grade	Grade Description
**0**	No apparent injury by light microscopy
**I**	Swelling of hepatocytes
**II**	Ballooning of hepatocytes
**III**	Lipid droplets in hepatocytes
**IV**	Necrosis of hepatocytes

### 3.6. Molecular docking

The active site regions of three enzymes, specifically NADPH oxidase, butyrylcholinesterase, and tyrosinase, allowed for the binding of the seven major compounds. As shown in (Table 4), all compounds exhibited satisfactory binding scores when docked with the three selected targets.

**Table 4:** The docking scores of the seven major compounds against the three enzymes, NADPH oxidase, BChE and tyrosinase.

Compound name	NADPH oxidase (Kcal/Mol)	Butyrylcholinesterase BChE (Kcal/Mol)	Tyrosinase (Kcal/Mol)
5,7-Dihydroxy-2’-methoxy-3’,4’-(methylenedioxy)isoflavone	-12.02	-11.74	-14.49
Sinapic acid 3-O-glucoside	-12.66	-10.70	-14.21
Syringetin-3-O-glucoside	-15.37	-13.20	-12.71
Isorhamnetin	-11.53	-15.85	-13.85
Linarin	-14.41	-13.81	-18.94
Kaempferol-3,7-O-bis-α-L-rhamnoside	-15.06	-13.66	-10.62
Cyanidin rutinoside	-14.01	-18.12	-13.94

**Fig 5:**
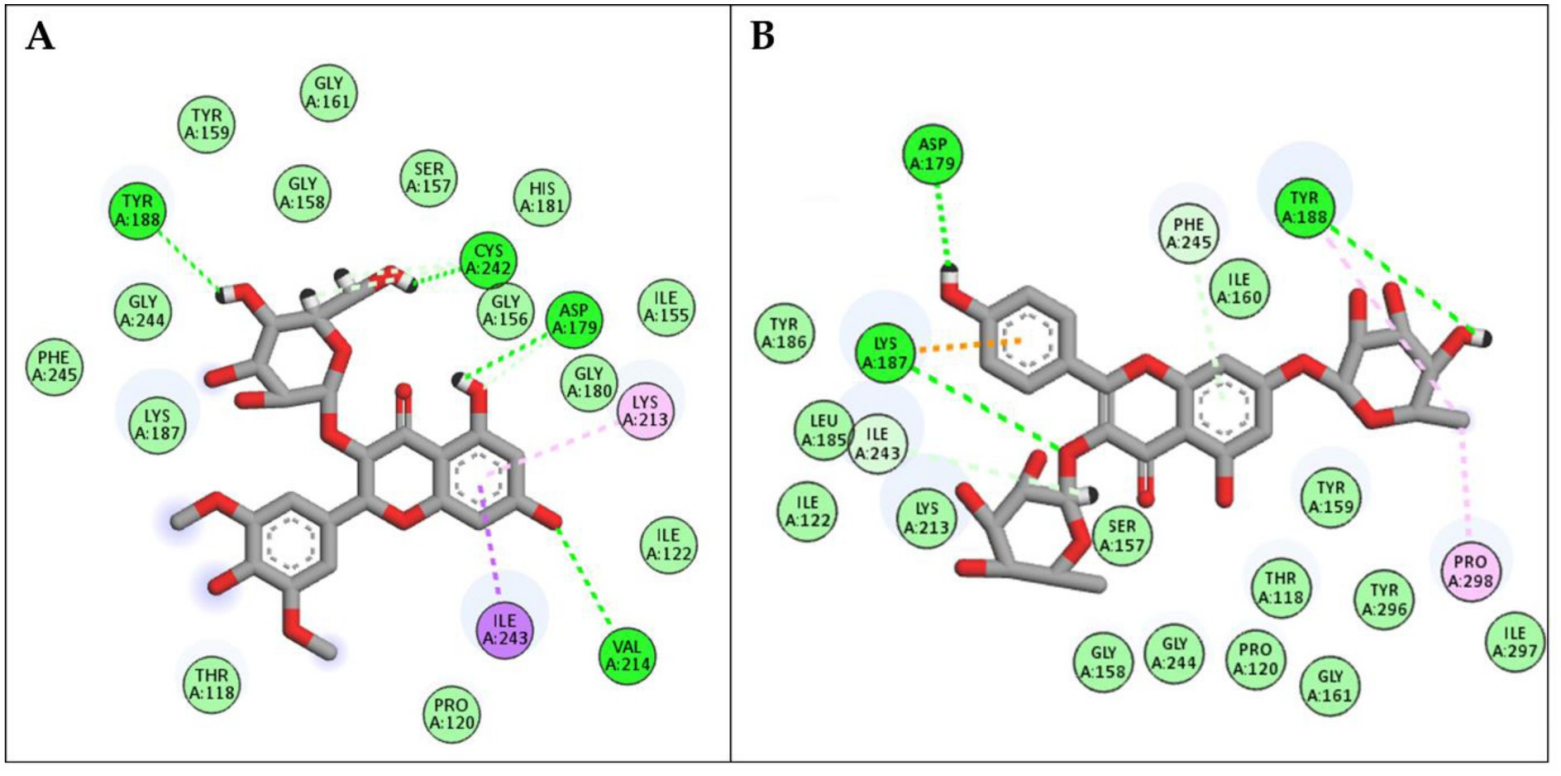
2D binding modes of (A) Syringetin-3-O-glucoside and (B) Kaempferol-3,7-O-bis-α-L-rhamnoside to the active binding sites of NADPH enzyme.

In the docking studies with NADPH oxidase, compounds syringetin-3-O-glucoside and kaempferol-3,7-O-bis-*α*-L-rhamnoside achieved the highest docking scores of -15.37 and -15.06 Kcal/mol, respectively. As seen in (Fig 5), syringetin-*O*-glucoside interacted with NADPH oxidase residues through hydrogen bond interactions with Asp179, Tyr188, Val214, and Cys242, in addition to various hydrophobic interactions with Lys213, Cys242, and Ile243. Similarly, Kaempferol-3,7-O-bis-*α*-L- rhamnoside interacted with Asp179, Lys187, Tyr188, Ile243, Phe245 and Pro298 through both hydrophilic and hydrophobic interactions.

**Fig 6:**
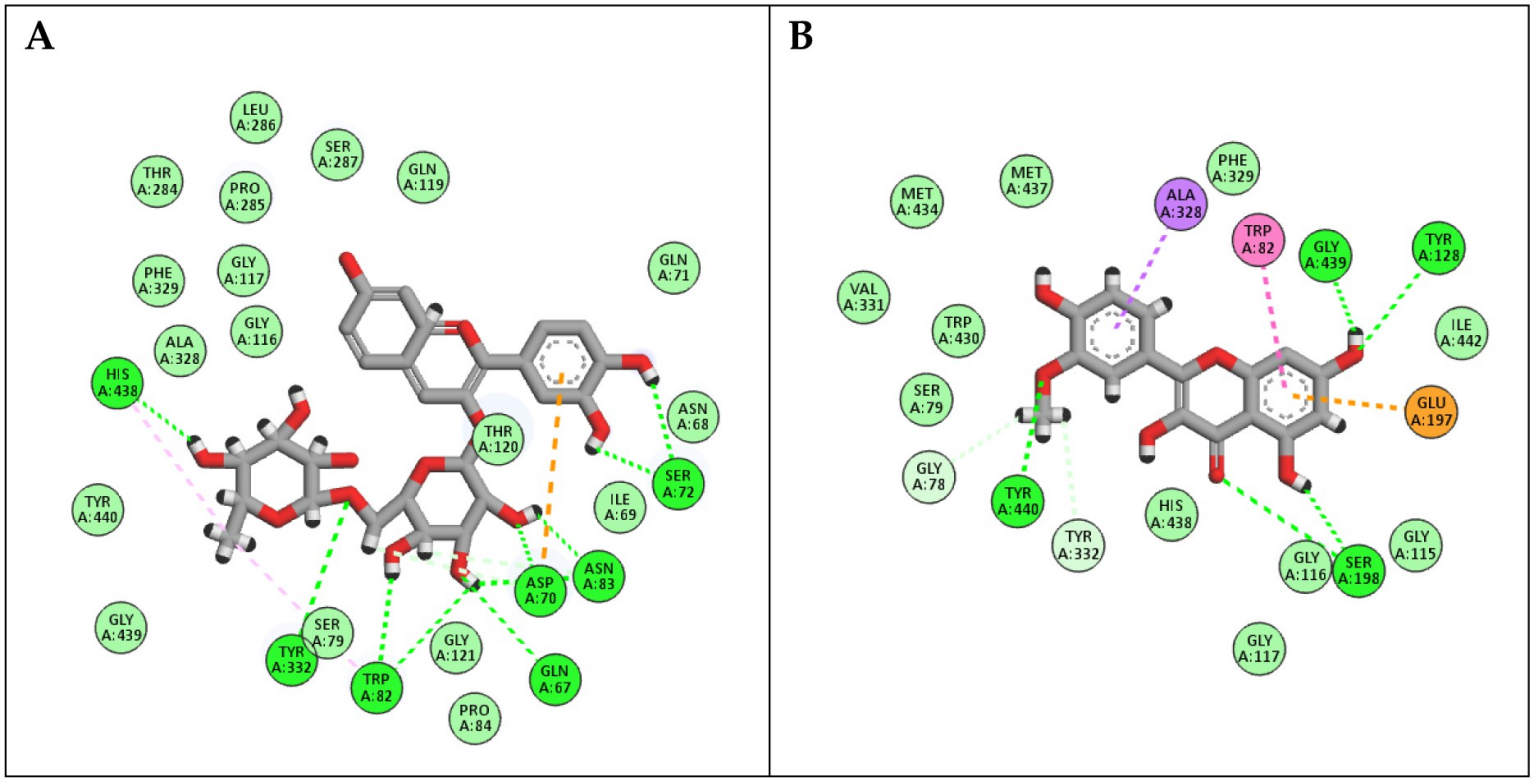
2D binding modes of (A) Cyanidin rutinoside and (B) Isorhamnetin to the active binding sites of BChE enzyme.

The docking investigations with BChE revealed that cyanidin rutinoside and isorhamnetin were the most efficient molecules, with docking scores of -18.12 and -15.85 Kcal/mol, respectively. As depicted in (Fig 6), cyanidin rutinoside was able to interact with BChE oxidase residues through hydrogen bond interactions with Gln67, Asp70, Ser72, Trp82, Asn83, Tyr332, and His438, in addition to various hydrophobic interactions with Asp70, Trp82, Asn83, and His438. In a similar way, isorhamnetin interacted with Gly78, Trp82, Tyr128, Glu178, Ser198, Ala328, Try332, Gly439 and Tyr440 through both hydrophilic and hydrophobic interactions.

**Fig 7:**
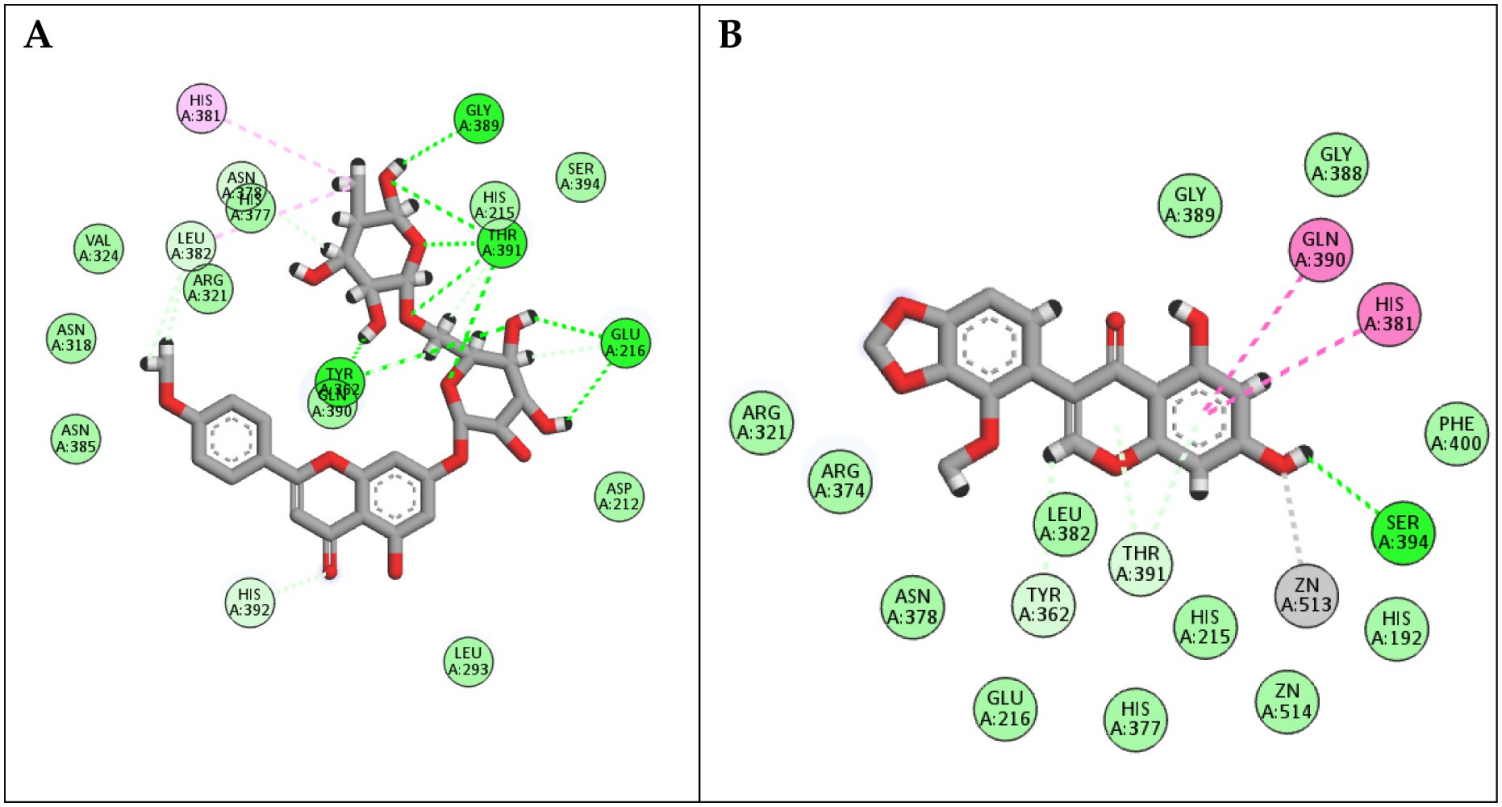
2D binding modes of (A) Linarin and (B) 5,7-Dihydroxy-2’-methoxy-3’,4’-(methylenedioxy) isoflavone to the active binding sites of Tyrosinase enzyme.

In the docking studies with tyrosinase, linarin and 5,7-dihydroxy-2’-methoxy-3’,4’-(methylenedioxy) isoflavone achieved docking scores of -18.92 and -14.49 Kcal/mol, respectively. As shown in (Fig 7) reveals, linarin could engage with tyrosinase active sites through forming hydrogen bond interactions with Glu216, Tyr362, Gly389, and Thr391; it formed various hydrophobic interactions with Glu216, His381, Leu382, Gln390, and Thr391. In the same way, 7-Dihydroxy-2’-methoxy-3’,4’-(methylenedioxy) isoflavone interacted with tyrosinase residues through both hydrophilic and hydrophobic interactions with His381, Gln391, Thr391, Ser394, and Zn513. In conclusion, all the compounds in the major extract demonstrated excellent docking scores with the three targets, suggesting the synergetic contribution of the whole extract.

## 4. Conclusions

Based on HPLC-ESI-MS/MS, the methanol extract of *M*. *sinaica* has been investigated and identified a total of 13 secondary metabolites, which included flavonoids, anthocyanins, and phenolic acids such as syringetin, kaempferol, and apigenin derivatives. It exhibited high total phenolics and flavonoid contents that were directly associated with its antioxidant capabilities as measured by the DPPH and FRAP assays. Moreover, MSLE exhibited noteworthy hepatoprotective activities and ameliorated the harmful effects of paracetamol toxicity in rats. The findings suggest that *M*. *sinaica* could be regarded as a plant of biological significance and a promising candidate for antioxidant and hepatoprotective agents.

## Supporting information

S1 Fig Calibration curve of evaluation of antioxidant activity using DPPH scavenging. (DOCX)

S2 Fig Calibration curve of evaluation of antioxidant activity using FRAP scavenging. (DOCX)

S3 Fig Total ion chromatogram (TIC) for *M*. *sinaica* methanol extract using HPLC/ESI/MS in the positive and negative ion mode. (DOCX)

S1 Graphical abstract (DOCX)
