# Supplementary figures and images for: Exploring the phytochemicals, antioxidant properties, and hepatoprotective potential of Moricandia sinaica leaves against paracetamol-induced toxicity: Biological evaluations and in Silico insights
Source: PLoS One. 2024 Oct 9;19(10):e0307901. doi: 10.1371/journal.pone.0307901 (PMC11463746; doi:10.1371/journal.pone.0307901)

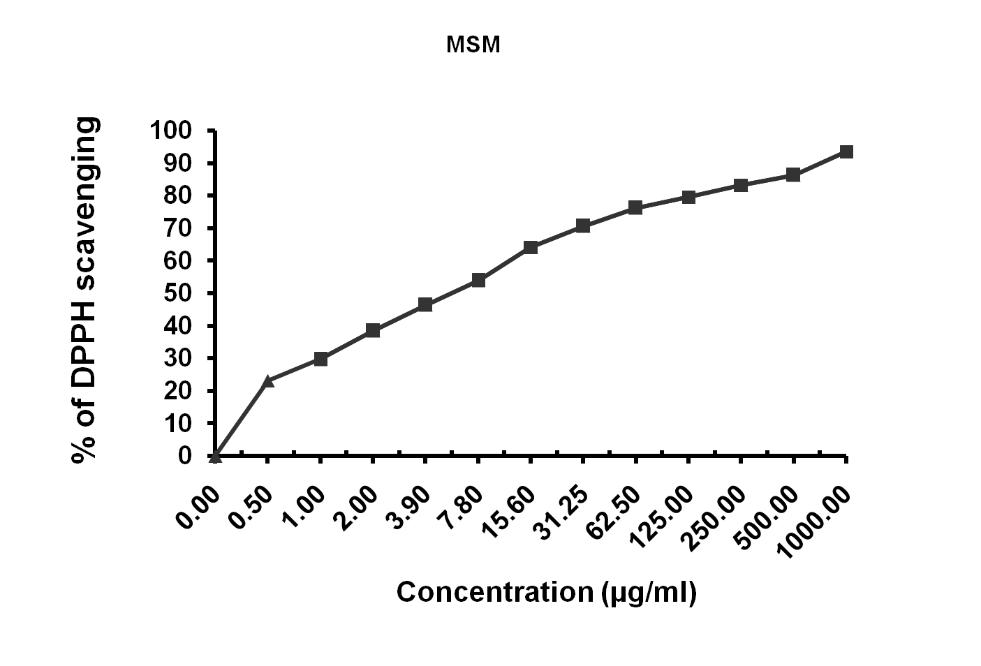


**Fig S1.** Calibration curve of Evaluation of Antioxidant Activity using DPPH scavenging

Supplement: S1 Fig — (DOCX) [file pone.0307901.s001.docx]

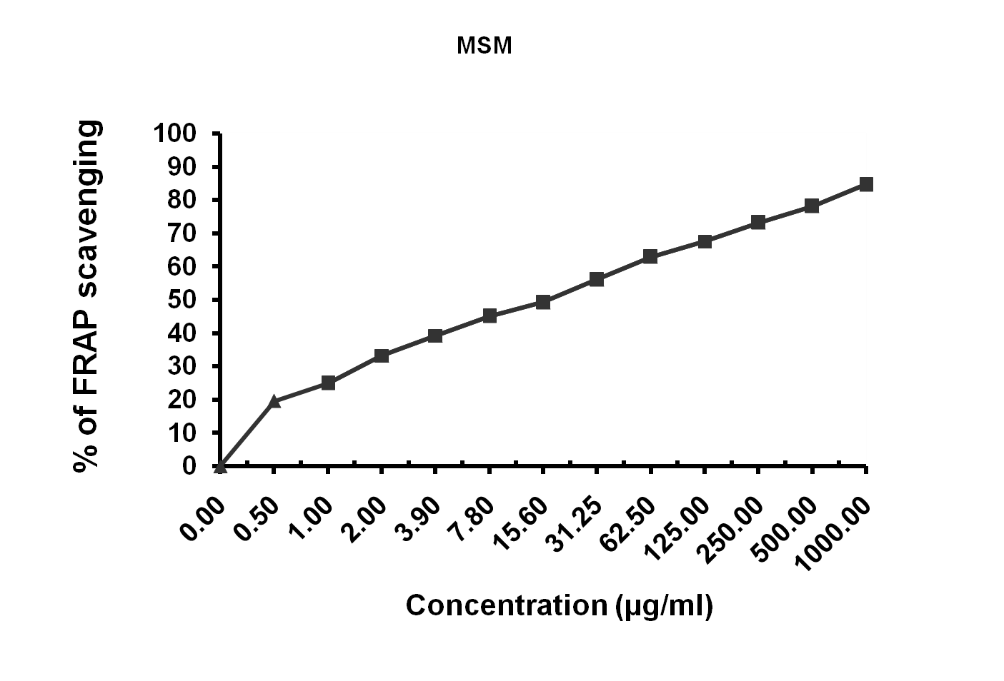


**Fig S2.** Calibration curve of Evaluation of Antioxidant Activity using FRAP scavenging.

Supplement: S2 Fig — (DOCX) [file pone.0307901.s002.docx]

**Graphical abstract**


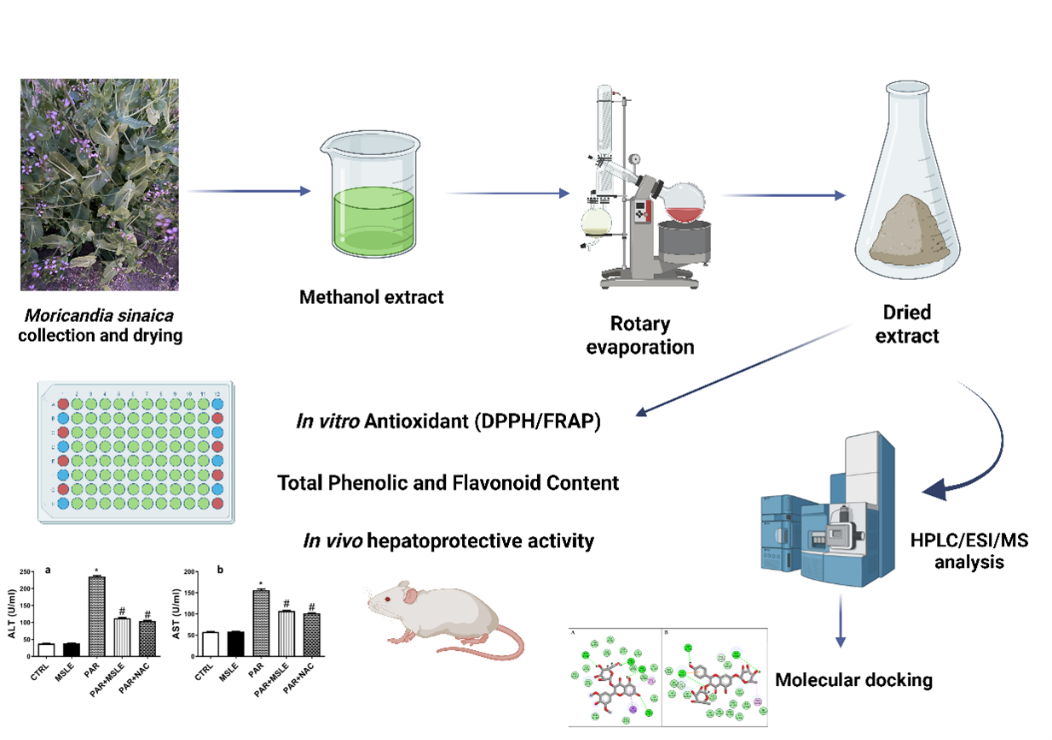

Supplement: S1 Graphical abstract — (DOCX) [file pone.0307901.s004.docx]
