# Supplementary material for: Exploring the phytochemicals, antioxidant properties, and hepatoprotective potential of Moricandia sinaica leaves against paracetamol-induced toxicity: Biological evaluations and in Silico insights
Source: PLoS One. 2024 Oct 9;19(10):e0307901. doi: 10.1371/journal.pone.0307901 (PMC11463746; doi:10.1371/journal.pone.0307901)
Supplement: S3 Fig — (DOCX) [file pone.0307901.s003.docx]

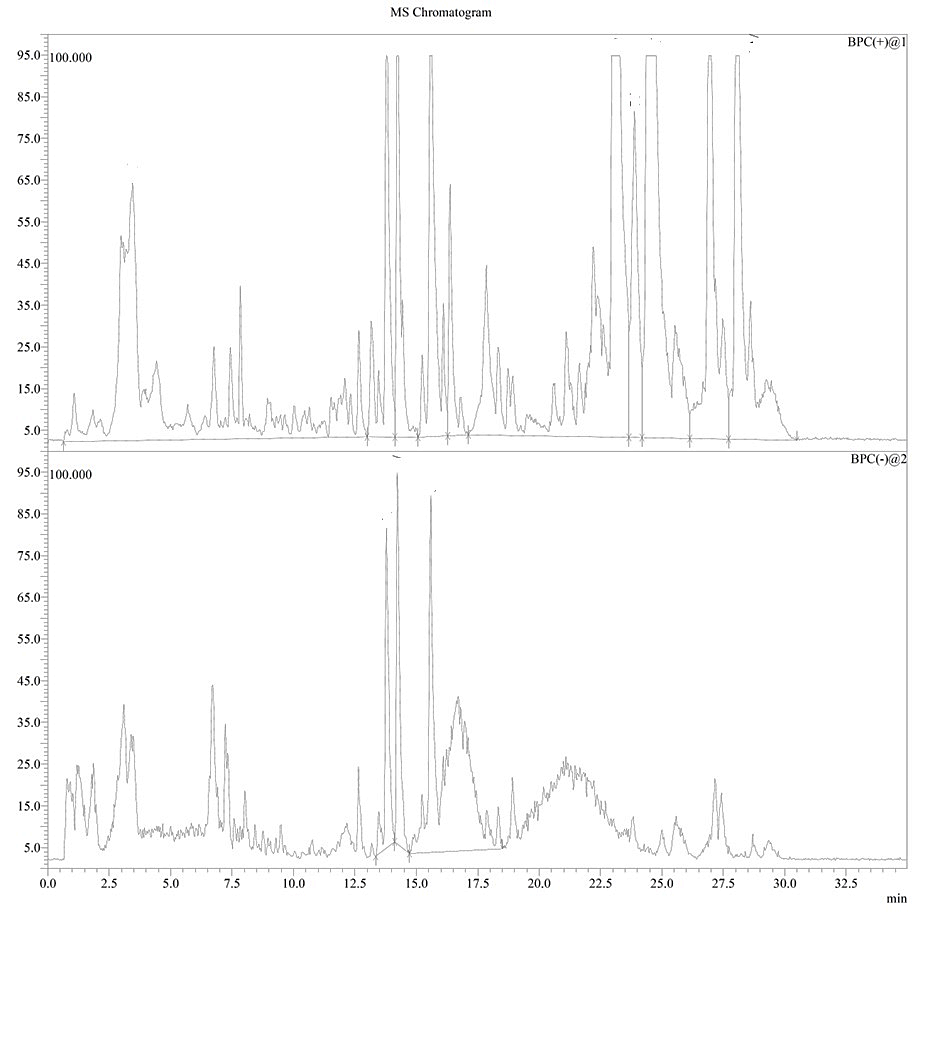


**Fig S3.** Total ion chromatogram (TIC) for *M. sinaica* methanol extract using HPLC/ESI/MS in the positive and negative ion mode.
